# Mechanical thrombectomy for a 12-year-old boy with acute ischemic stroke: A case report

**DOI:** 10.1097/MD.0000000000021436

**Published:** 2020-07-24

**Authors:** Yuchai Huang, Zhen Wang, Changluo Li, Ning Ding

**Affiliations:** a Emergency Department of Changsha Central Hospital; b Neurology Department of Changsha Central Hospital, Changsha, Hunan Province, China.

**Keywords:** acute ischemic stroke, cerebral infarction, children, mechanical thrombectomy

## Abstract

**Rationale:**

Acute ischemic stroke (AIS) is one of the most severe diseases that endanger human health. It is very common among middle-aged and elderly people, but it is rare in children. The treatment varies among children and adults, since the cause for AIS in children differs from that in adults. In adults with AIS, endovascular therapy has been recommended, but guidelines for endovascular therapy in children with AIS have not been established yet. In China, few relevant evidence is present so far in clinical research of mechanical thrombectomy in the treatment for children with AIS.

**Patient concerns:**

A 12-year-old boy without any special physical collision and trauma was admitted to emergency department of Changsha central hospital due to hemiplegia of left limbs for 3 hours.

**Diagnoses:**

He was diagnosed with AIS after magnetic resonance imaging (MRI) examination and magnetic resonance angiography (MRA) examination. Cerebral infarction in the right parietal, temporal, insular, and frontal lobes was revealed by the MRI test. The MRA test detected occlusions in right internal carotid artery, A1 segment of right anterior cerebral artery, right middle cerebral artery, and distal branch.

**Interventions:**

Mechanic thrombectomy and antiplatelet aggregation therapy with clopidogrel helped the patient to recover, along with active rehabilitation training.

**Outcomes:**

A significant improvement in muscle strength of his left limbs was proved. He walked by himself and had 2 of Modified Rankin Scale (MRS). At 1-year follow-up visit, he recovered well except feeling a bit pain of left lower limb when walking, with finally MRS of 1.

**Conclusions:**

Mechanical thrombectomy can be performed safely for children with AIS, but needs a further research with large samples.

## 1 Introduction

Acute ischemic stroke (AIS) is comparably rare in children. It is reported that the annual morbidity of AIS in children was approximate 2 to 6/100,000 in the United States.^[1,2]^ In Hong Kong, the incidence of pediatric stroke was estimated as about 2.1 cases per 100,000 children-years.^[3]^ In children with AIS, the mortality was 3% to 6% and 25% experienced recurrent strokes,^[4]^ whereas more than half of pediatric stroke survivors might suffer from cognitive impairment and long-term physical disabilities.^[5]^ However, in China, it has not yet been statistically proved in the morbidity of children with AIS.

In adults with AIS, endovascular therapy has been recommended for more than 1 decade,^[6]^ which revolutionized the management of AIS and significantly improved outcomes for patients. Guidelines for endovascular therapy in pediatric stroke have not been established yet. In China, few relevant evidence is present so far in clinical research of endovascular therapy in the treatment for children with AIS.

In the present case, a 12-year-old boy with AIS successfully recovered after the treatment of mechanical thrombectomy along with active rehabilitation training. To the best of our knowledge, a successful case of mechanical thrombectomy treatment in children with AIS was firstly documented in Hunan province of China.

## 2 Case report

**Figure 1:**
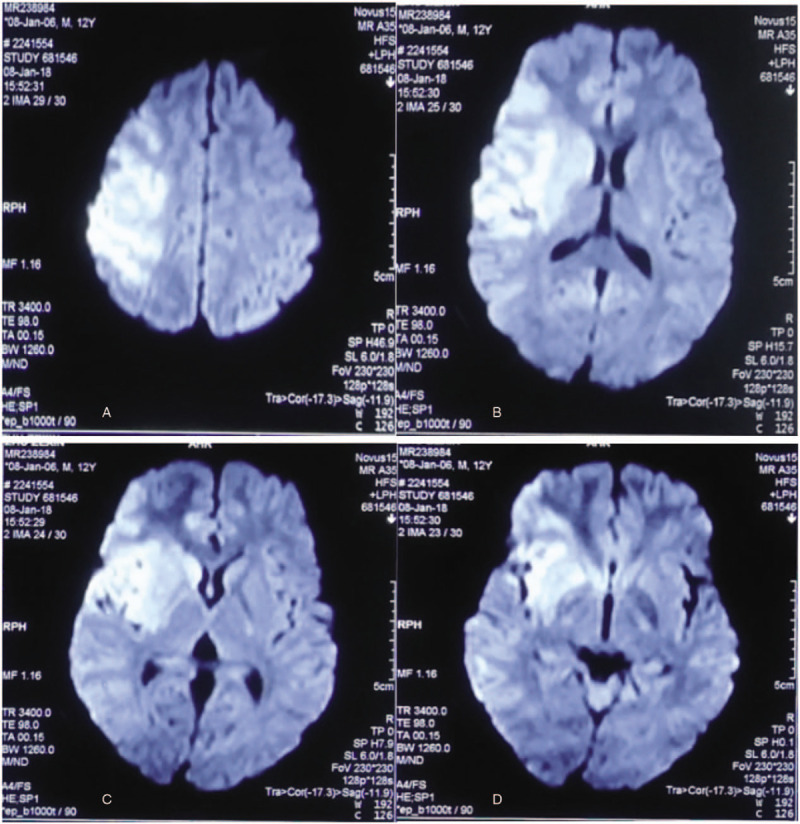
Brain magnetic resonance imaging (MRI; January 8, 2018). Acute cerebral infarction in the right parietal (A), temporal (B,C), insular (B,C), and frontal lobes (D).

**Figure 2:**
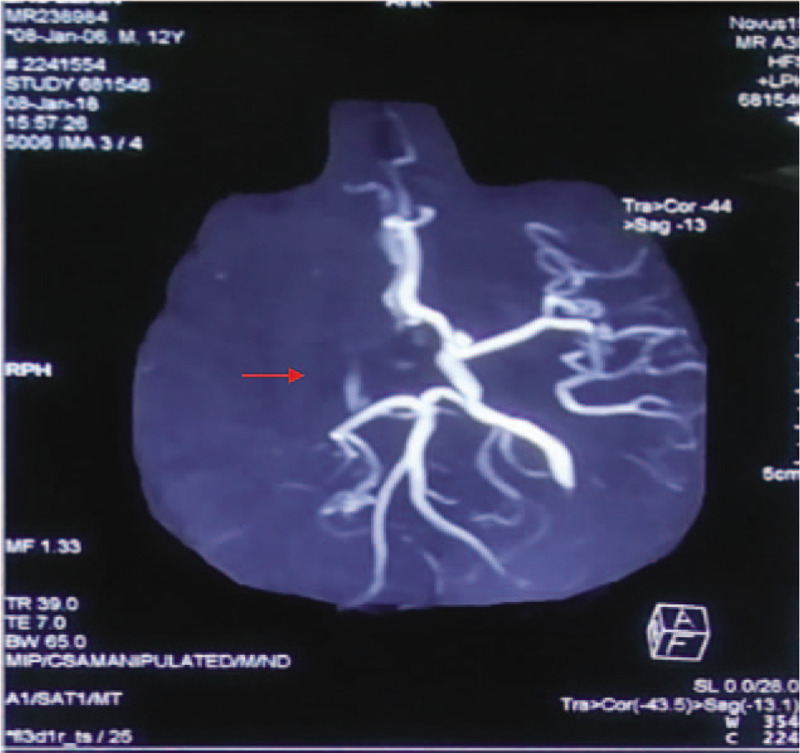
Brain magnetic resonance angiography (MRA; January 8, 2018). Occlusions in the right internal carotid artery, A1 segment of right anterior cerebral artery, right middle cerebral artery, and distal branches.

**Figure 3:**
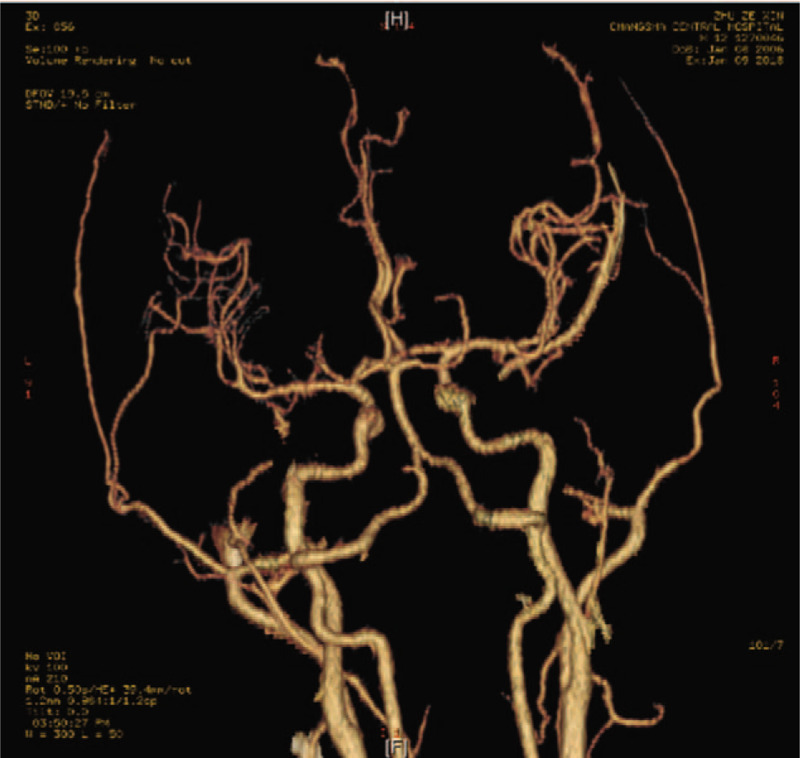
Brian computed tomography angiography (CTA; January 9, 2018). Blood flow of right internal carotid artery was restored, and the right middle cerebral artery and the anterior cerebral artery were demonstrated normally.

The 12-year-old boy was admitted to emergency department of Changsha Central Hospital at 17:45 on January 8, 2018, due to hemiplegia of left limbs for 3 hours without physical collision and trauma. Besides the symptom of hemiplegia, he also had urinary incontinence and unclear speech. He was diagnosed with AIS in a local hospital after performing a magnetic resonance imaging (MRI) examination (Fig. 1) and magnetic resonance angiography (MRA) examination (Fig. 2). Cerebral infarction in the right parietal, temporal, insular, and frontal lobes was revealed by the MRI test. The MRA test detected occlusions in right internal carotid artery, A1 segment of right anterior cerebral artery, right middle cerebral artery, and distal branch. For further treatment, he was admitted to our hospital. The patient reported no history of coagulation disorders, cardiovascular disease, special medication, or any other systemic immune disorder, nor was there any recent history of trauma. On admission, the boy was not mentally oriented. His weight was 65 kg and height 168 cm, with 36.3°C body temperature, 82 beats per minute pulse rate, 22 per minute breathing rate, and 119/62 mm Hg blood pressure. The physical examination revealed normal heartbeat and no crackles in the lungs. Neurological examination showed Glasgow Scale of 14, equal and reactive pupils, and unclear speech. In addition, both eyes gazed to the right side. He showed flattened left nasolabial fold, and left deviation of the protruded tongue. The muscle tension of left limbs increased while the muscle strength of left upper limb and left lower limb were grade 1 and grade 2, respectively. He could not perform the left finger-nose test and the heel-knee-tibia test and his left pathological reflex was positive with 12 points of National Institute of Health Stroke Scale. Laboratory findings demonstrated that a white blood cell count of 12,770/mm^3^, with 84.5% neutrophils as well as hemoglobin concentration of 12.6 g/dL and a platelet count of 297,000/mm^3^. There was no abnormality in liver and kidney function test. D-dimer was 0.10 μg/mL, and C-reactive protein was 3.03 mg/L. Triglyceride was 1 mmol/L and low-density lipoprotein was 2.29 mmol/L. Tests of anti-Sjogren's syndrome B, anti-Sjogren's syndrome A, anti-Jo1, antinuclear antibody, antihistone antibody (AHA) and anti-SM measured by enzyme-linked immunosorbent assay were negative. After admission, emergency cerebral angiography and mechanical thrombectomy were performed under local anesthesia. Cerebral angiography revealed that occlusion in the end segment of right internal carotid artery and normal blood flow of left anterior communicating artery. The operation of mechanical thrombectomy was performed successfully. For antiplatelet aggregation, tirofiban was given continuously intravenously (0.15 μg/kg/min body weight). After 24 hours, oral clopidogrel was prescribed (75 mg once per day) and atorvastatin (20 mg once per day) were also administered for him. And after 48 hours, tirofiban was discontinued while clopidogrel (75 mg once per day) and atorvastatin (20 mg once per day) were continuously given. On January 9, 2018, computed tomography angiography (Fig. 3) illuminated that the blood flow of right internal carotid artery was restored, and the right middle cerebral artery and the anterior cerebral artery were demonstrated normally. At the same time, further examinations were arranged for the young patient. Laboratory examinations including thyroid function, antineutrophil antibody, biomarkers of lupus, and tumor revealed no abnormality. Dynamic electrocardiogram showed sinus arrhythmia. There was no abnormality in Doppler echocardiography. Ultrasonography of carotid artery, abdomen, and veins of both lower extremities were also normal.

Around 2 weeks after admission, the patient was transferred to the rehabilitation department for further treatment. After being with active rehabilitation training for several months, physical examination for him revealed that muscle strength of left limbs were both grade 3, and clear speech. On May 5, 2018, he was discharged from hospital and prescribed with clopidogrel (75 mg once per day) and atorvastatin (20 mg once per day). The patient was followed up for a year. On June 20, 2018, the patient came to hospital for a checkup and the results showed a significant improvement in his physical conditions. Muscle strength of left limbs both increased to grade 4 and muscle tension also improved. He could walk by himself without any assistance and had Modified Rankin Scale (MRS) of 2. In June 2019, he recovered well except feeling a bit pain of left lower limb when walking, with final MRS of 1.

## 3 Discussion

Cerebral infarction, also known as ischemic stroke, is one of the most severe diseases that seriously endangers human health. It is caused by the stenosis or occlusion of the cerebral arteries, which leads to cerebral ischemic necrosis. It is common in middle-aged and elderly people. It has many risk factors^[7–11]^ including hypertension, diabetes, heart disease, atrial fibrillation, smoking, dyslipidemia, and carotid artery stenosis. Although it is rare in children, the mortality and disability rates are pretty high.^[12]^ The etiology of AIS in children is more complex compared to that in adults. The main causes in children are infection, trauma, heart disease, cerebrovascular malformation, vasculitis, thrombotic disease, and hereditary disease.^[13–18]^ Despite the fact that laboratory technique has been dramatically developed, the etiology of some cases is still not clear. This case had no history of trauma and special medication. No abnormalities were found after the relevant examinations such as connective tissue disease, thyroid disease, tumor, infection, thrombosis, blood lipid disorder, and heart dysfunction. The findings of blood vessels and thrombosis during the operation not only confirmed the simple thromboembolism, but also excluded vascular dissection and atherosclerosis. The limitation in this case is that thrombus pathology failed to examine the factors of vasculitis and Moyamoya’ disease.

The aim of AIS treatment is to restore cerebral blood circulation, to save ischemic penumbra, to alleviate secondary neuronal damage, and to improve the degree of neurological deficit. Great progress has been made in treatment and long-term prevention of ischemic stroke in adults. There is sufficient clinical evidence to confirm the effectiveness of intravenous thrombolysis and mechanical thrombectomy in treatment for adults with AIS.^[19–21]^ However, there is still lack of sufficient clinical evidence of intravenous thrombolysis and mechanical thrombectomy in the treatment and prevention of AIS in children. At present, the 2018 AHA/ASA Guidelines for Early Management of AIS recommends intravenous thrombolysis and endovascular therapy are available for adults older than 18 years,^[22]^ whereas controversy still exists in children with AIS. The effect and risk of endovascular therapy in children with AIS is still unclear. Previous research demonstrated that about 2% of pediatric stroke in the United States were treated with intravenous thrombolysis,^[23]^ whereas some studies also suggested that the family members of the children should be totally informed concerning the uncertainty of the safety and effectiveness of intravenous thrombolysis for children.^[14,24,25]^

In the present case, the child who had no infection, trauma, congenital heart disease, and rheumatic diseases, was surprisingly diagnosed of AIS after an imaging examination. It clearly indicated that the right middle cerebral artery and the right internal carotid artery were occluded. For the occlusion of internal carotid artery and middle cerebral artery, the latest guidelines for the diagnosis and treatment of AIS pointed out that the rate of recanalization by intravenous thrombolysis alone was low in adults and mechanical thrombectomy should be recommended.^[22]^ In this case, the 12-year-old teenage patient had adult's height and weight. After a considerate discussion with his family members, we did mechanical thrombectomy for him. An increasing number of reports illuminated that endovascular therapy for children with AIS may be also safe and effective.^[25–27]^ A retrospective, multicenter cohort study including 73 children with AIS demonstrated that 87% of the patients treated with thrombectomy had a significant improvement of neurologic disability, which proved that the safety of mechanical thrombectomy in pediatric patients was the same as in adults.^[25]^ Another research about 68 pediatric patients revealed that mechanical thrombectomy group had better neurological outcome and clinical prognosis with fewer complications compared to intravenous thrombolysis group.^[26]^ Even one case reported that a 2-year-old male patient with AIS was performed for mechanical thrombectomy successfully and recovered well.^[27]^ However, there was still very low incidence of complications such as cerebral hemorrhage and orolingual angioedema in some cases,^[28,29]^ and the treatment of children with AIS needs to be further explored with large samples.

In conclusion, the underlying causes for AIS in the pediatric population are different from those in adults. So far, no guidelines have been available for the treatment in the children with AIS. In this case, the child with AIS has successfully recovered after mechanical thrombectomy, which provided clinical reference for the diagnosis and treatment of children with AIS in China. Further study is needed to help establishing guidelines for pediatric stroke as soon as possible.^[30]^

## Author contributions

**Conceptualization:** Zhen Wang, Changluo Li.

**Investigation:** Yuchai Huang, Ning Ding.

**Writing – original draft:** Yuchai Huang.

**Writing – review & editing:** Ning Ding.
